# Research trends on endoscopic therapy for non-variceal upper gastrointestinal bleeding: a bibliometric analysis from 1991 to 2024

**DOI:** 10.1097/JS9.0000000000001907

**Published:** 2024-07-03

**Authors:** Ren-Chun Du, Li-Xiang Ling, Yu-Xin Hu, Yu-Tian Xiao, Yan-An Zhou, Yi Hu

**Affiliations:** a Department of Gastroenterology, Digestive Disease Hospital, The First Affiliated Hospital of Nanchang University, Nanchang, Jiangxi Province; b Department of Surgery, The Chinese University of Hong Kong, Shatin NT, Hong Kong, People’s Republic of China

## Introduction

Highlights

- This is the first bibliometric analysis assessing the research trends and hotspots of endoscopic therapy for non-variceal upper gastrointestinal bleeding (NVUGIB).
- The USA was the most productive and cooperative country, followed by China and Japan, and The Chinese University of Hong Kong was the most productive institution.
- Peptic ulcers or erosions were the most common etiologies, and injection or spray were the most common hemostatic protocols, followed by hemoclips or band ligation and thermal coagulation.
- With the development of endoscopic devices, the hemostasis rate generally increased and the 30-day rebleeding rate, mortality, and surgery intervention rate decreased. The initial hemostasis rate, definite hemostasis rate, 30-day rebleeding rate, mortality, surgery intervention rate, and days in hospital from 2021 to 2024 were 97.0%, 89.7%, 7.1%, 3.5%, 1.2%, and 7.4, respectively.

Non-variceal upper gastrointestinal bleeding (NVUGIB) is characterized as hemorrhaging occurring proximal to the ligament of Treitz in the absence of ectatic veins that typically form in the esophagus, stomach, or proximal duodenum. Endoscopic treatment is considered the first therapeutic approach in the management of hemorrhage and serves to reduce the occurrence of persistent or recurrent bleeding^1^. With the development of prospective studies evaluating the impact of early endoscopy, a variety of endoscopic techniques have emerged (e.g. over-the-scope clips and sprayed hemostatic powder), leading to the increased efficacy in the treatment of NVUGIB^2–5^.

Bibliometric analysis is a statistical technique employed to identify the research focus and trends within a specific field^6,7^. In this study, we aimed to investigate the research trends and hotspots focusing on endoscopic therapy and its application in the management of NVUGIB using a quantitative method to obtain an overview of this field and provide guidance on the treatment of endoscopy for NVUGIB patients.

## Methods

A search was conducted in the Web of Science database to retrieve relevant studies using a combination of the search terms ‘endoscopy’ and ‘NVUGIB’. All relevant publications were independently evaluated by two authors in two stages and any differences and conflicts were discussed and resolved by the third author. The following information was recorded: title, author, institution, country, publication, citation, H-index, etiology and hemostatic type, success rate, rebleeding rate, and mortality rate after treatment. VOSviewer software and GraphPad Prism were employed to analyze and visualize the data.

## Results

### Analysis of authors, institutions, and countries

**Figure 1:**
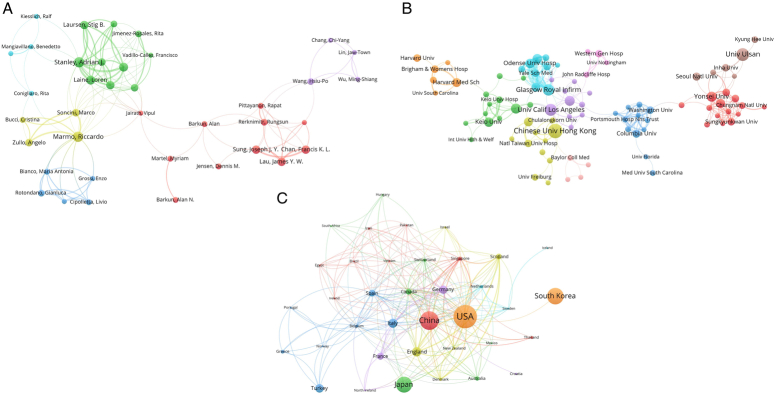
The cooperation network of authors (A), institutions (B), and countries (C).

As shown in Supplementary Figure S1 (Supplemental Digital Content 1, http://links.lww.com/JS9/D8), a total of 703 studies meeting the inclusion criteria of this study were included. The network map of the authors is shown in Figure 1A. A total of 38 authors and 6 clusters are displayed. Lee JH reported the highest number of publications of articles (*n*=15), and Sung JJY possessed the highest number of citations (*n*=1299) (Supplementary Table S1, Supplemental Digital Content 2, http://links.lww.com/JS9/D9). The cooperation network of the institutions involved in the publications is presented in Figure 1B. The Chinese University of Hong Kong possessed the highest number of publications (*n*=18) and citations (*n*=1526) (Supplementary Table S2, Supplemental Digital Content 2, http://links.lww.com/JS9/D9). The global distribution of publications and international collaborations among 37 countries and 211 cooperations were shown in Figure 1C, and the analysis of the top 10 countries in terms of their publications, total citations, and H-index was presented in Supplementary Table S3 (Supplemental Digital Content 2, http://links.lww.com/JS9/D9). The United States had the highest number of publications (*n*=123), citations (*n*=3263), and H-index (*n*=33).

### Research trends on etiology and hemostatic therapy of NVUGIB

**Figure 2:**
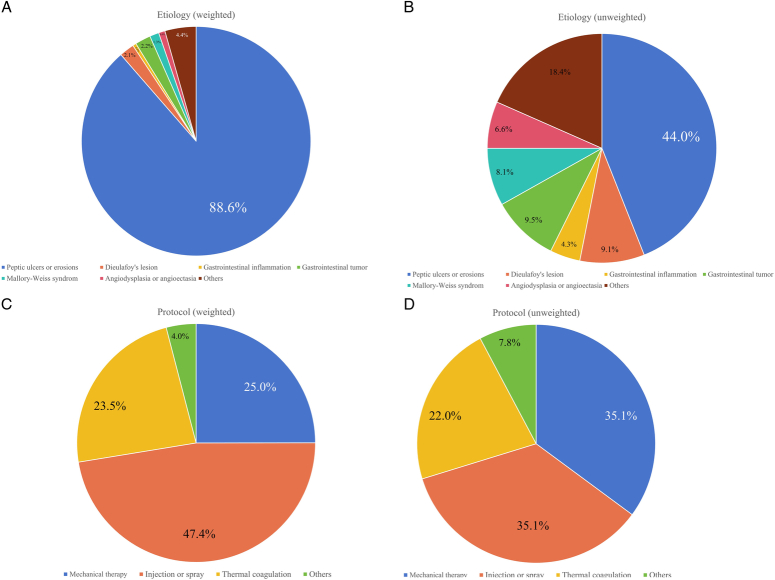
The proportion of different types of weighted (A) and unweighted (B) etiology; the proportion of different types of weighted (C) and unweighted (D) hemostatic protocol.

As illustrated in Figure 2A, peptic ulcers were identified as the predominant cause of NVUGIB, accounting for 88.6% of cases. Additionally, in terms of research output, peptic ulcers represented the most studied topic, constituting 44.0% of the published papers, followed by gastrointestinal tumors (9.5%) and Dieulafoy’s lesions (9.1%) (Fig. 2B). Injections or sprays were the most commonly used methods, accounting for 47.4% of the hemostatic therapies, followed by hemoclips or band ligations (25.0%) and thermal coagulation (23.5%) (Fig. 2C). Moreover, injection, spray, hemoclips, and band ligations were used in 70.2% of the studies, and thermal coagulation accounted for 22.0% of the total publications (Fig. 2D).

### Temporal variation in the effectiveness of endoscopic therapy for NVUGIB

As shown in Table 1, over these years, the initial hemostasis rate has shown a gradual increase, while the surgical intervention rate has significantly decreased from an original 9.9% to the current 1.2%. Specifically, in Asia and Europe, the surgical intervention rates dropped from an original 11.3% and 9.0% to the current 2.0% and 0.5%, respectively. The rebleeding rates in the USA and Europe, as well as the mortality rates in Asia, also showed decreased trends. From 2021 to 2024, the recorded rates were as follows: initial hemostasis at 97.0%, definite hemostasis at 89.7%, 30-day rebleeding at 7.1%, mortality at 3.5%, surgical intervention at 1.2%, and average hospital stay at 7.4 days.

**Table 1:** The outcome (hemostasis rate, rebleeding rate, mortality, surgery intervention rate, and days in hospital) of endoscopic therapy for non-variceal upper gastrointestinal bleeding from 1991 to 2024.

	Outcome					
Region/year	Initial hemostasis rate	Definitive hemostasis rate	Rebleeding rate	Mortality	Surgery	Days in hospital
Asia						
1991–1995	94.3%	84.2%	13.5%	5.0%	11.3%	6.0
1996–2006	95.8%	86.9%	11.0%	4.8%	9.0%	5.5
2007–2015	95.7%	87.7%	8.5%	4.6%	6.4%	6.1
2016–2024	96.2%	88.9%	7.3%	4.1%	2.0%	8.0
USA and Europe						
1991–1995	93.1%	86.3%	14.9%	4.3%	9.0%	11.9
1996–2006	95.5%	88.1%	10.4%	3.9%	3.6%	8.9
2007–2015	98.3%	91.6%	8.4%	1.7%	2.4%	10.1
2016–2024	98.7%	90.6%	8.3%	2.3%	0.5%	7.6
All						
1991–1995	93.7%	85.3%	14.2%	4.6%	9.9%	6.7
1996–2000	95.0%	87.8%	8.3%	5.9%	9.0%	4.7
2001–2005	95.2%	88.8%	9.9%	3.8%	3.4%	9.6
2006–2010	95.8%	89.9%	9.0%	2.2%	4.2%	7.3
2011–2015	96.2%	88.3%	8.7%	2.5%	3.8%	7.6
2016–2020	96.8%	90.9%	11.4%	3.9%	3.0%	10.0
2021–2024	97.0%	89.7%	7.1%	3.5%	1.2%	7.4

## Discussion

To the best of our knowledge, this is the first bibliometric analysis assessing the research trends and hotspots of endoscopic therapy for NVUGIB, and the main findings were presented as follows: (1) USA was the most productive country with a total publication of 123, followed by China (99) and Japan (82); (2) The Chinese University of Hong Kong was the most productive institution and Sung JJY, Chan FKL, and Lau JYW were the most influential authors; (3) peptic ulcers and erosions were the most common etiologies, accounting for 88.6% of the total etiology and injection or spray were the common hemostatic protocols (47.4%); (4) with the development of endoscopic devices, the hemostasis rate increased, rebleeding rate, mortality and surgery intervention rate decreased and the initial hemostasis rate, definite hemostasis rate, 30-day rebleeding rate, mortality, surgery intervention rate and days in hospital from 2021 to 2024 were 97.0%, 89.7%, 7.1%, 3.5%, 1.2%, and 7.4, respectively.

This study had several limitations. First, the search was limited to the Web of Science core collection database, which may have resulted in the exclusion of relevant literature from other databases, potentially affecting the comprehensiveness of the findings. Second, the exclusion of studies that did not use specific keywords like ‘endoscopy’ and ‘NVUGIB’ may have led to the omission of relevant studies. Third, the number of studies is limited; as such we cannot divide the regions into specific countries. Fourth, there is a possibility of bias in the study, which may have influenced the results.

In conclusion, owing to the remarkable advancements in the field of novel endoscopic devices such as the novel application of over-the-scope clips and hemostatic powder, the hemostasis rate generally increased and the 30-day rebleeding rate, mortality, and surgery intervention rate decreased, while more randomized clinical trials are still needed to optimize the endoscopic technology for NVUGIB.

## Ethical approval

Not applicable.

## Consent

Not applicable.

## Source of funding

This study was supported by the National Natural Science Foundation of China (Nos 82000531 and 82360118); the Project for Academic and Technical Leaders of Major Disciplines in Jiangxi Province (No. 20212BCJL23065); the Key Research and Development Program of Jiangxi Province (No. 20212BBG73018); the Key Laboratory Project of Digestive Diseases in Jiangxi Province (2024SSY06101); National Science and Technology Award Reserve Cultivation Project (20192AEI91008); the First Affiliated Hospital of Nanchang University Clinical Research and Cultivation Project (YFYLCYJPY202002).

## Author contribution

R.-C.D. and L.-X.L.: performed the literature search; Y.-T.X. and Y.-A.Z.: collected the data; R.-C.D., L.-X.L. and Y.-X.H.: performed the statistical analysis and wrote the manuscript; Y.H.: designed the study and revised the manuscript. All authors contributed to the article and approved the final manuscript.

## Conflicts of interest disclosure

The authors declare no conflicts of interest.

## Research registration unique identifying number (UIN)

Not applicable.

## Guarantor

Yi Hu.

## Data availability statement

The data that support the findings of this study are available from the corresponding author upon reasonable request.

## Provenance and peer review

Not commissioned, externally peer-reviewed.

## Supplementary Material

## References

[1] Barkun AN Bardou M Martel M. Prokinetics in acute upper GI bleeding: a meta-analysis. Gastrointest Endosc 2010;72:1138–1145. 20970794 10.1016/j.gie.2010.08.011

[2] Binmoeller KF Thonke F Soehendra N. Endoscopic hemoclip treatment for gastrointestinal bleeding. Endoscopy 1993;25:167–170. 8491134 10.1055/s-2007-1010277

[3] Schmidt A Gölder S Goetz M. Over-the-scope clips are more effective than standard endoscopic therapy for patients with recurrent bleeding of peptic ulcers. Gastroenterology 2018;155:674–686.e6. 29803838 10.1053/j.gastro.2018.05.037

[4] Toka B Eminler AT Karacaer C. Comparison of monopolar hemostatic forceps with soft coagulation versus hemoclip for peptic ulcer bleeding: a randomized trial (with video). Gastrointest Endosc 2019;89:792–802. 30342026 10.1016/j.gie.2018.10.011

[5] Baracat FI de Moura DTH Brunaldi VO. Randomized controlled trial of hemostatic powder versus endoscopic clipping for non-variceal upper gastrointestinal bleeding. Surg Endosc 2020;34:317–324. 30927124 10.1007/s00464-019-06769-z

[6] Du RC Ouyang YB Hu Y. Research trends on artificial intelligence and endoscopy in digestive diseases: a bibliometric analysis from 1990 to 2022. World J Gastroenterol 2023;29:3561–3573. 37389238 10.3748/wjg.v29.i22.3561 PMC10303508

[7] Zhang J Luo Z Zhang R. The transition of surgical simulation training and its learning curve: a bibliometric analysis from 2000 to 2023. Int J Surg 2024;110:3326–3337. 38729115 10.1097/JS9.0000000000001579 PMC11175803

