# Supplementary figures and images for: Research trends on endoscopic therapy for non-variceal upper gastrointestinal bleeding: a bibliometric analysis from 1991 to 2024
Source: Int J Surg. 2024 Jul 3;111(1):1473–6. doi: 10.1097/JS9.0000000000001907 (PMC11745770; doi:10.1097/JS9.0000000000001907)

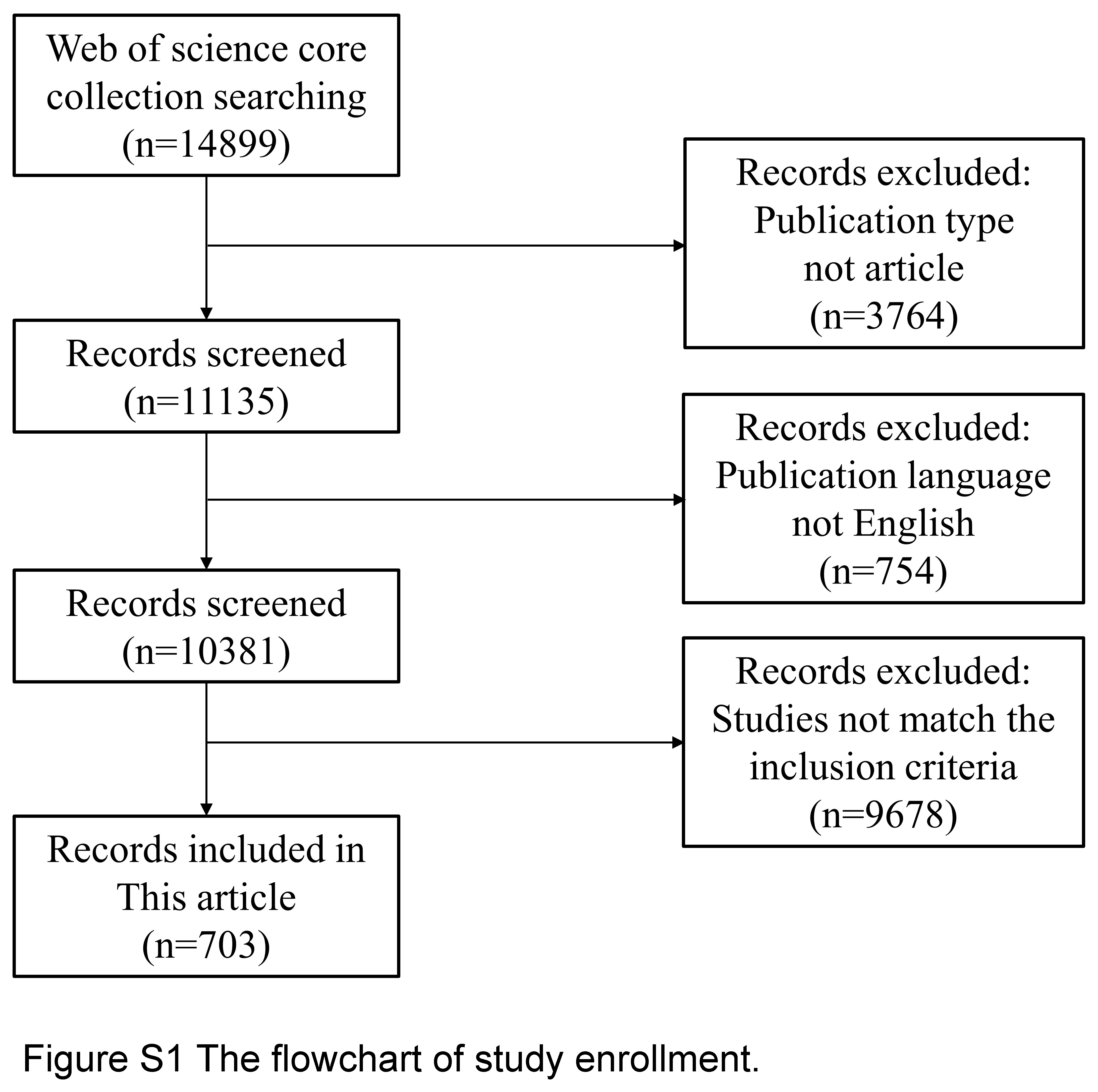

Supplement: Supplementary file 1 [file js9-111-1473-s001.jpg]
