# Supplementary material for: Research trends on endoscopic therapy for non-variceal upper gastrointestinal bleeding: a bibliometric analysis from 1991 to 2024
Source: Int J Surg. 2024 Jul 3;111(1):1473–6. doi: 10.1097/JS9.0000000000001907 (PMC11745770; doi:10.1097/JS9.0000000000001907)
Supplement: Supplementary file 2 [file js9-111-1473-s002.docx]

**Table S1 The top 10 most productive authors in the field of endoscopic therapy for non-variceal upper gastrointestinal bleeding from 1991 to 2024**

| **Authors** | **Publications** | **Citations** | **H-Index** |
| --- | --- | --- | --- |
| Lee JH | 15 | 208 | 10 |
| Marmo R | 14 | 468 | 9 |
| Lau JYW | 12 | 1059 | 10 |
| Kim JH | 12 | 154 | 10 |
| Song HJ | 11 | 69 | 6 |
| Stanley AJ | 11 | 468 | 9 |
| Sung JJY | 10 | 1299 | 10 |
| Chan FKL | 10 | 1168 | 9 |
| Rotondano G | 10 | 429 | 10 |
| Saltzman JR | 10 | 400 | 9 |

**Table S2 The top 10 most productive institutions in the field of endoscopic therapy for non-variceal upper gastrointestinal bleeding from 1991 to 2024**

| **Affiliations** | **Publications** | **Citations** | **H-Index** |
| --- | --- | --- | --- |
| Chinese University of Hong Kong | 18 | 1526 | 13 |
| Harvard University | 18 | 537 | 12 |
| Assistance Publique Hopitaux Paris | 18 | 355 | 9 |
| University of California System | 18 | 470 | 11 |
| US Department of Veterans Affairs | 16 | 598 | 10 |
| Veterans Health Administration | 16 | 598 | 10 |
| Universite Paris Cite | 15 | 311 | 8 |
| University of Ulsan | 15 | 142 | 8 |
| Harvard Medical School | 14 | 407 | 10 |
| Mayo Clinic | 14 | 276 | 8 |

**Table S3 The top 10 most productive countries in the field of endoscopic therapy for non-variceal upper gastrointestinal bleeding from 1991 to 2024**

| **Countries** | **Publications** | **Citations** | **H-Index** |
| --- | --- | --- | --- |
| USA | 123 | 3263 | 33 |
| China | 99 | 2278 | 30 |
| Japan | 82 | 1271 | 18 |
| Korea | 81 | 1064 | 16 |
| England | 43 | 1417 | 20 |
| Italy | 40 | 1168 | 20 |
| Germany | 38 | 1140 | 18 |
| Turkey | 37 | 442 | 12 |
| France | 28 | 611 | 13 |
| Spain | 28 | 752 | 16 |
